# Recent Advances in the Biosynthesis of Carbazoles Produced by Actinomycetes

**DOI:** 10.3390/biom10081147

**Published:** 2020-08-05

**Authors:** Masaya Kobayashi, Tomohisa Kuzuyama

**Affiliations:** 1 Biotechnology Research Center, The University of Tokyo, 1-1-1 Yayoi, Bunkyo-ku, Tokyo 113-8657, Japan; 60green60@gmail.com; 2 Graduate School of Agricultural and Life Sciences, The University of Tokyo, 1-1-1 Yayoi, Bunkyo-ku, Tokyo 113-8657, Japan

**Keywords:** biosynthesis, carbazoles, cyclization, enzymes, protein structure

## Abstract

Structurally diverse carbazole alkaloids are valuable due to their pharmaceutical properties and have been isolated from nature. Experimental knowledge on carbazole biosynthesis is limited. The latest development of in silico analysis of the biosynthetic gene clusters for bacterial carbazoles has allowed studies on the biosynthesis of a carbazole skeleton, which was established by sequential enzyme-coupling reactions associated with an unprecedented carbazole synthase, a thiamine-dependent enzyme, and a ketosynthase-like enzyme. This review describes the carbazole biosynthetic mechanism, which includes a key step in enzymatic formation of a tricyclic carbazole skeleton, followed by modifications such as prenylation and hydroxylation in the skeleton.

## 1. Introduction

**Figure 1:**
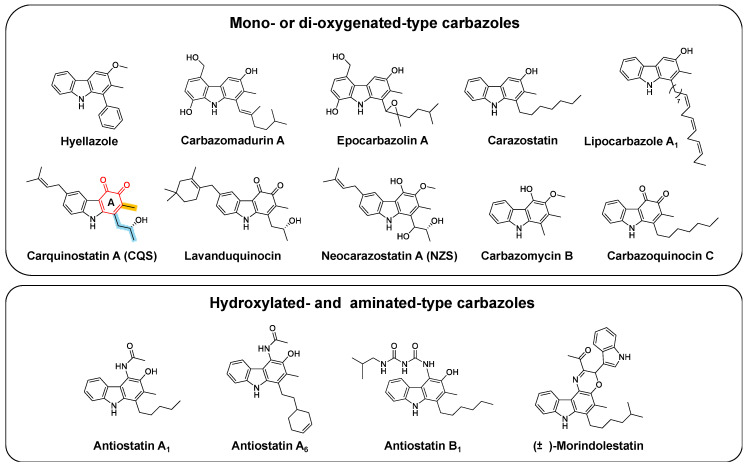
Structures of bacterial carbazole natural products. The carbazole skeletons are classified into “mono- or di-oxygenated-type” and “hydroxylated- and aminated-type”. Ring A of the carbazole moiety (red), *meta*-methyl group (orange), and *para*-alkyl side chain (cyan) are indicated in carquinostatin A (CQS).

Carbazole, a tricycle consisting of two benzene rings fused on either side of a pyrrole core, was first isolated from coal tar in 1872 [1]. Later, murrayanine, the first naturally occurring carbazole alkaloid discovered, was identified as an antibiotic from the curry tree (*Murraya koenigii*) in 1965 [1]. Since then, carbazole alkaloids have been considered important molecules due to their biological activities and structural diversity. Heterocyclic molecules with a carbazole system possess an indole-like structure with a benzene ring fused onto the 2,3-positions of an indole ring, and a large π-conjugated backbone that can afford chemical features such as electrophilic aromatic substitution, oxidative reactions, and alkylation reactions, which generate a variety of modified derivatives concomitant with biological activities [2]. These compounds have been isolated from various organisms, including higher plants, fungi, and some bacteria [1]. Although the primary sources of carbazole alkaloids are plant-based derivatives, carbazole skeletons that contain *meta*-methyl groups, *para*-alkyl side chains, and/or aromatic ring oxygenations have been isolated from microorganisms and marine sources (Figure 1). Based on the oxidative status of ring A, the types of carbazole skeletons can be classified into two main categories: “mono- or di-oxygenated-type” and “hydroxylated- and aminated-type”. In 1979, 3-oxygenated tricyclic carbazoles, hyellazoles [3], were isolated from the marine cyanobacterium *Hyella caespitosa*. Carbazomadurins A and B [4], epocarbazolins [5], carazostatin [6], and lipocarbazoles [7], which all possess a 3-oxygenated carbazole skeleton, were identified in microorganisms. Antiostatins A1-6 and B2-5 [8,9] and (±)-morindolestatin [9] are carbazole derivatives with an acetamide group or a substituted urea chain. Carquinostatins A (CQS) and B [10,11,12], lavanduquinocin [13], neocarazostatins A–C [14], carbazoquinocins A–F [15], and carbazomycins A–H [16,17,18,19] were isolated in the early 1990s and are representatives of carbazole-3,4-quinone alkaloids. This group of bacterial carbazoles shows potential as antioxidants to protect neuronal cells against oxidative damage caused by free radicals. Their pharmaceutical potential provides an opportunity to discover and develop medicine for a variety of diseases, such as ischemia-reperfusion, atherosclerosis, inflammation, autoimmune disease and cancer initiation [20,21]. In fact, edaravone, a free radical scavenger (but not a carbazole derivative), is used in the clinical treatment of oxidative stress in neurodegenerative processes, including amyotrophic lateral sclerosis (ALS) [22].

## 2. Elucidation of the Carbazole Biosynthetic Pathway

### 2.1. Discovery of the Carbazole Synthase CqsB2

**Figure 2:**
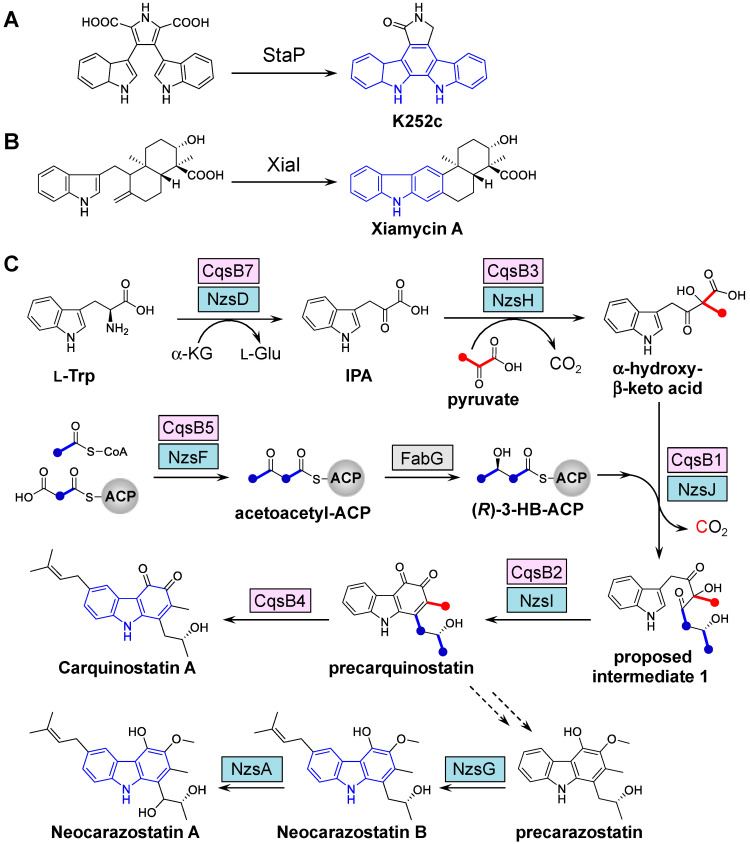
Biosynthesis of carbazole skeleton. (**A**) StaP for indolocarbazole biosynthesis. (**B**) XiaI for xiamycin biosynthesis. (**C**) Biosynthetic pathways of carquinostatin A and neocarazostatin A. The carbon atoms derived from pyruvate and acetate are indicated in red and blue, respectively. The CqsB enzymes for carquinostatin A and the corresponding Nzs enzymes for neocarazostatin A are shown in pink and cyan, respectively.

Only a small number of enzymes that catalyze the cyclization necessary for the synthesis of the carbazole skeleton have been found in nature, including StaP involved in indolocarbaozle biosynthesis [23] and XiaI involved in xiamycin biosynthesis (Figure 2) [24,25]. StaP, which is a member of the cytochrome P450 family, catalyzes the formation of the indolocarbazole core by intramolecular aryl–aryl coupling and oxidative decarboxylation in staurosporine biosynthesis. XiaI catalyzes a flavin-dependent oxidative cyclization reaction, tailoring indolosesquiterpene biosynthesis. Some carbazole alkaloids possess *ortho*-quinone or a similar oxygenated-aromatic ring (Figure 1), and the majority of these compounds are obtained from *Actinomycetales* bacteria. The carbazole metabolites produced by bacteria have been investigated by several tracer experiments, which have indicated that the carbazole skeleton is derived from tryptophan, pyruvate, and two molecules of acetate [26,27,28]. As the indole moiety of the carbazole skeleton is derived from tryptophan, the condensation reaction between tryptophan or its derivative and pyruvate is necessary in the early step of carbazole skeleton biosynthesis. Huang and coworkers performed biosynthetic studies of bacterial carbazoles and were the first to identify the neocarazostatin A (NZS) biosynthetic gene cluster in *Streptomyces* sp. MA37 through genome mining and gene inactivation [29]. In their pioneering studies, they describe the functions of five key enzymes, including the phytoene synthase-like prenyltransferase NzsG [29], the P450 hydroxylase NzsA [29], the thiamine diphosphate (ThDP)-dependent enzyme NzsH [30], a free-standing acyl carrier protein (ACP) NzsE [31], and the classical β-ketoacyl-ACP synthase (KAS) III NzsF [31]. Biochemical assays demonstrated that NzsG and NzsA mediate sequential carbazole prenylation and hydroxylation, respectively, in the last two steps leading to the production of NZS (see Section 3). In addition, further experiments provided biochemical evidence that three other enzymes (NzsH, E, and F) are responsible for the assembly of the carbazole skeleton of NZS [30,31]. NzsH, a ThDP-dependent enzyme, catalyzes an acyloin condensation reaction between indole-3-pyruvate (IPA) and pyruvate to generate a β-ketoacid intermediate. In vivo genetic experiments and RT-PCR analysis also indicated that the *nzsH* gene is essential for the biosynthesis of NZS [30]. NzsE and NzsF catalyze a decarboxylative condensation between acetyl-CoA and the malonyl-ACP (NzsE-bound malonyl thioester) to generate acetoacetyl-NzsE. Since NzsF can only accept NzsE as its cognate ACP substrate, it can be assumed that NzsE and NzsF constitute pathway-specific KAS III enzyme pairs for the assembly of NZS [31]. In their studies, however, the biosynthetic machinery associated with carbazole nucleus formation through an unexpected cyclization remained unclear.

To gain insights into the cyclization mechanism in carbazole nucleus formation, biosynthetic studies of the CQS produced by *S. exfoliatus* 2419-SVT2 were conducted [32]. CQS, which has a carbazole skeleton similar to that of NZS, possesses an *ortho*-quinone function and a prenyl moiety on the carbazole nucleus. Based on the heterologous production of CQS and gene deletion analysis, as well as biochemical analysis, the complete biosynthetic pathway of CQS was established and the carbazole synthase CqsB2 (NzsI for NZS biosynthesis) was functionally characterized (Figure 2). The unprecedented enzyme CqsB2 is responsible for the cyclization of the acyl side chain moiety on the unstable intermediate to form the *ortho*-quinone-containing A ring of the carbazole intermediate (precarquinostatin), which is a key step in carbazole biosynthesis. As the CqsB2-catalyzed reaction product consists of the carbazole tricyclic ring with *ortho*-quinone, a *meta*-methyl group, and a *para*-alkyl side chain, the carbazole intermediate may be a common key precursor of the two main types of bacterial carbazole alkaloids listed in Figure 1.

**Figure 3:**
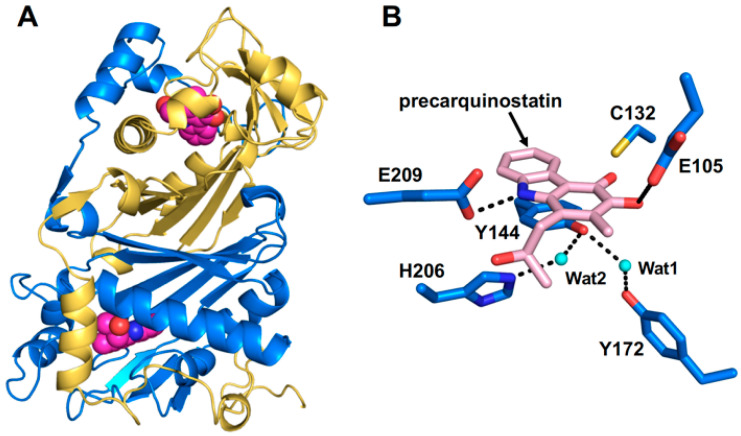
(**A**) Homodimer structure of CqsB2. (**B**) Active site of CqsB2 bound with precarquinostatin.

**Figure 4:**
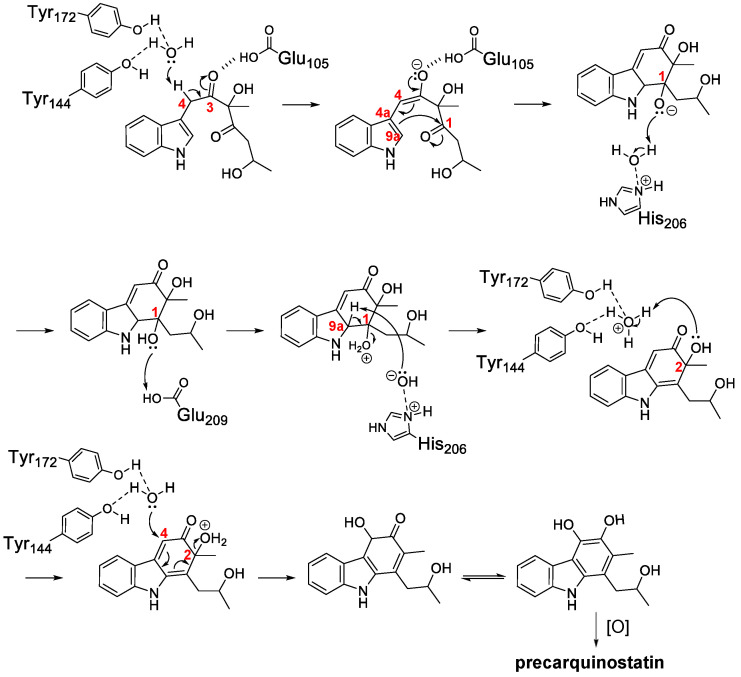
Proposed CqsB2-catalyzed cyclization reaction. The positions of the carbon atoms described in the text are numbered in red.

Based on the crystal structures of CqsB2 and mutagenesis-based biochemical assays, a possible catalytic mechanism for oxidative cyclization was proposed for the CqsB2-catalyzed formation of the carbazole skeleton [32]. The proposed biological assembly of CqsB2 is a dimer structure, which is structurally similar to the type II polyketide aromatase/cyclases (ARO/CYCs) such as TcmN and WhiE-ORFIV in type II polyketide biosynthesis despite their low sequence identity to CqsB2 (Figure 3) [33,34]. ARO/CYCs belong to the Bet v1-like superfamily, the members of which possess a deep interior pocket that binds hydrophobic ligands [35]. Of those enzymes, ARO/CYCs possess a path from the external environment to the interior of the active site pocket, which is necessary to accommodate the linear poly-β-keto intermediate conjugated with ACP. In contrast, the entrance of the active-site cleft of CqsB2 is narrow because the pocket is covered with the α1 helix of the N-terminal arm of the other subunit [32]. The substrate of CqsB2 is unstable indole intermediate **1**, which is assembled from IPA and (*R*)-3-hydroxybutyryl (HB)-ACP through sequential condensation and alkylation reactions catalyzed by CqsB3 and CqsB1, respectively. In the active site pocket of CqsB2, the Tyr144- and Tyr172-coordinated water molecule (Wat1) may act as a general base to abstract a proton from C4 to form the enolate at the carbonyl oxygen of C3 (Figure 4). Glu105 may also promote C4 proton abstraction and stabilize the negatively charged enolate at C3. These interactions could facilitate the attack of the C1 carbonyl by C4a–C9a π-electrons to form a new ring. Then, a water molecule interacting with His206 and a side-chain carboxy group of Glu209 may donate protons for the oxyanion. Following abstraction of the proton at C9a by the His206-coordinated water molecule (Wat2), C1–C9a double bond formation and dehydration at C1 could occur. Next, a water molecule (Wat1) coordinated to Tyr144 and Tyr172 may donate a proton to the C2 hydroxy group. A water molecule, probably Wat1, could attack the C4 carbon to form aromatic ring A with concomitant elimination of water from C2 and the formation of a catechol-containing intermediate, which is spontaneously oxidized to an *ortho*-quinone-containing carbazole.

The in vitro reconstitution of three enzymes in the NZS biosynthetic studies, NzsJ, NzsI, and NzsH (corresponding to CqsB1, CqsB2, and CqsB3, respectively), together with other necessary substrates, was also reported for the assembly of the A ring moiety of NZS [36]. Furthermore, isotopic labeling studies with H_2_^18^O in NZS biosynthesis demonstrated that the hydroxyl group at C4 of ring A originates from one water molecule [36]. The biosynthesis of “di-oxygenated-type” or “hydroxylated- and aminated-type” carbazoles could be facilitated by methylation or transamination after the formation of the catechol moiety of ring A. In the case of “mono-oxygenated-type” carbazoles, hydride-based nucleophilic attack may occur at C4 during the aromatization of ring A instead of water molecules. However, the biosynthetic machinery for the hydride-based assembly of the A ring moiety of “mono-oxygenated-type” carbazoles remains to be identified. CqsB2 and NzsI show high sequence identity (80%) to each other and therefore catalyze the same reaction to generate a key carbazole intermediate (precarquinostatin). In addition, intriguingly, CqsB2 and NzsI do not require any cofactors. Further biochemical experiments and enzyme kinetic parameters of CqsB2 and NzsI for investigation of the unprecedented cofactor-free cyclization mechanism remain to be established to date due to their unstable substrate (intermediate **1** in Figure 2C). Contrary to CqsB2 and NzsI, StaP, a member of the cytochrome P450 family, is a heme-containing enzyme that catalyzes a reaction in staurosporine biosynthesis by means of an indole radical cation intermediate, and XiaI is a flavin-dependent enzyme that catalyzes oxidative cyclization in indolosesquiterpene biosynthesis.

### 2.2. Reconstitution of Carbazole Backbone

Tracer experiments and biochemical analysis revealed that the backbone of the carbazole ring is derived from tryptophan, pyruvate, and 3-HB-ACP [26,27,28]. Biochemical assays identified four enzymes for carbazole synthesis in NZS biosynthesis, including the ThDP-dependent enzyme NzsH (CqsB3 homolog) [30], the ACP NzsE (CqsB6 homolog), the classical β-ketoacyl-ACP synthase III NzsF (CqsB5 homolog, KASIII), and FabG homolog in fatty acid biosynthesis [31]. On the other hand, the total biosynthetic pathway of the CQS was established by biochemical characterization of the five CQS biosynthetic gene products CqsB1, 2, 3, 6, and 7 [32].

**Figure 5:**
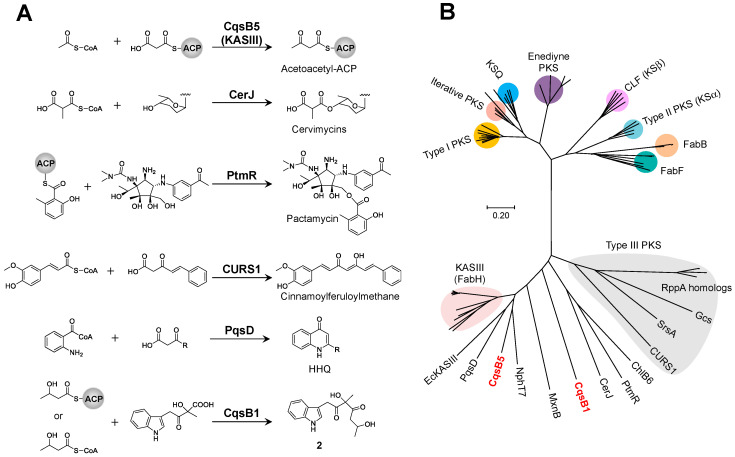
(**A**) The promiscuous condensation reactions catalyzed by KASIII-like enzymes. (**B**) The phylogenetic relationship of ketosynthases. The tree was calculated using the neighbor-joining method by MEGA7 [47]. Accession numbers: CqsB1 (BF24923.1) in red, CqsB5 (BBF24927.1) in red, NphT7 (D7URV0.1), CerJ (AEI91069.1), ChlB6 (AAZ77679.1), PtmR (ACJ24876.1), MxnB (AGS77282.1), PqsD (P20582.2), EcKASIII (P0A6R0.1), FabH (AAQ08929.1, AAC18104.1, CAM58805.1, ACI88883.1, Q54206.1, Q9F6D4.1, P72392.1, AAV84077.1, WP_012382088.1, WP_003969377.1, WP_003979735.1), FabB (YP_001881145.1, NP_416826.1, ZP_04562837.1, ZP_00134992.2, AAA99449.1), FabF (NP_645683.1, NP_344945.1, YP_143679.1, NP_415613.1, WP_011028323.1, YP_143679.1); type I PKS [AmphA (AAK73512.1), AveA1 (BAC68648.1), OlmA1 (BAC70610.1), PikAI (Q9ZGI5.1), RevA (BAK64649.1), Lsd11 (BAG85026.1), MonAI (ANZ52459.1), 8,8a-deoxyoleandolide synthase 1 (AAF82408.1:1062–1474, AAF82408.1:2548-2971)]; iterative type I PKS [Azi26 (ABY83164.1), PctS (BAF92601.1), PokM1 (ACN64831.1), AviM (X55776.1), ChlB1 (AAZ77673.1)]; enediyne type I PKS [AerE (AAO25864.1), EspE (AAP92148.1), PksE (AAO25904.1), SgcE (ANY94470.1)]; KSα [ActIORF1 (NP_629237.1), EncA (AAF81728.1), OxyA (AAZ78325.1), GrhA (AAM33653.1)]; KSβ [(TcmL (AAA67516.1), Snoa2 (CAA12018.1), ActIORF2 (CAA45044.1), AknC (AAF70107.1), SimA2 (AAK06785.1)]; type III PKS [RppA (WP_011027653.1), RppA (WP_012382077.1), RppA (WP_003970937.1), RppA (EFD70720.1), RppA (WP_078524272.1), Gcs (NP_631277.1), CURS1 (C0SVZ6.1), SrsA (BAG17301.1)]; KSQ [ChmGI (AAS79459.1), ChlA1 (AAZ77693.1), ConA (AAZ94386.1), GfsA (BAJ16467.2), HlsA (BAF02921.1)].

The initial step of precarquinostatin assembly in CQS biosynthesis is deamination, catalyzed by the aminotransferase CqsB7, which converts L-tryptophan to IPA (Figure 2). The C–C bond formed between IPA and pyruvate to yield α-hydroxy-β-keto acid is catalyzed by the ThDP-dependent enzyme CqsB3. The carbazole-synthesizing reaction involves the KASIII-like enzyme CqsB1 and carbazole synthase CqsB2. CqsB1 catalyzes the decarboxylative condensation of an α-hydroxy-β-keto acid with 3-HB-ACP to form unstable indole intermediate **1**, followed by the CqsB2-catalyzed formation of ring A of the carbazole skeleton. Biochemical studies on CQS biosynthesis clearly established that precarquinostatin is synthesized from α-hydroxy-β-keto acid through sequential alkylation and cyclization reactions catalyzed by CqsB1 and CqsB2, respectively. The CqsB1-catalyzed reaction product (which is indole intermediate **1**) was spontaneously converted to indole-containing esters because of the instability of the product. The NzsJ (CqsB1 homolog)-catalyzed reaction was analyzed, and structurally similar isomers of indole-acetyl ester were also identified [36]. This report led to the speculation that intermediate **1** is the true product of NzsJ that underwent a spontaneous α-ketol rearrangement reaction. Similar observations of intramolecular rearrangement were confirmed in the interconversion from the dibenzo[*b*]fluorene skeleton to a benzo[*g*]chromene and the biogenetic interconversions between prekinamycin and isoprekinamycin [37]. Density-functional theory (DFT) calculations demonstrated that the structure of intermediate **1** actually has higher energy than the rearranged products spontaneously produced from intermediate **1**. LC-MS analysis showed that the proposed intermediate **1** did not accumulate and could undergo rearrangement immediately. These results suggested that the concerted action of CqsB1 and CqsB2 is crucial for controlling aromatic carbazole ring formation to suppress spontaneous intramolecular rearrangement of unstable indole intermediate **1**. The type II polyketide ARO/CYC domain, which acts in a “chaperone-like” manner, helps direct nascent polyketide intermediates into particular reaction channels [38]. In the absence of ARO/CYC, the highly reactive poly-β-keto acyl chains undergo spontaneous aldol reactions, generating shunt products with various chain lengths and cyclization patterns [39]. Similar to ARO/CYC, CqsB2 may serve as a scaffold for carbazole assembly by stabilizing the conformation of the unstable substrate. CqsB1 and NzsJ are the first identified KASIII-like enzymes that catalyze the condensation of a β-hydroxy acyl side chain and an indole-containing derivative in carbazole alkaloid biosynthesis. In contrast, the KASIII-like enzymes RkD, PqsD, CURS1, CerJ, and PtmR catalyze a variety of reactions (Figure 5A) [40,41,42,43,44]. Of these KASIII-like enzymes, CerJ [43] and PtmR [44] exhibit a broad-range substrate specificity to generate various natural product analogs. It should be noted that CqsB1, PqsD, and CURS1 may catalyze decarboxylative condensation of a β-keto acid with an ACP- or CoA-bound substrate using a similar mechanism as that for the normal decarboxylative condensation of malonyl-ACP with acetyl-CoA by typical KASIII. To investigate the relationship and diversity of KASIII-like enzymes, the phylogeny of a subset of the ketosynthase family, including CqsB1 and CqsB5, was examined (Figure 5B). Since homologous KASIII proteins relate to type III polyketide synthases (PKSs), each subfamily forms a large clade. Considering that CqsB1 and CqsB5 belong to the KASIII subfamily that functions in fatty acid biosynthesis, these enzymes may have evolved from this primary metabolism.

Regarding the supply of 3-HB-ACP, investigations on NzsF, a classical KASIII, revealed that FabG (NADPH-dependent reductase) for fatty acid biosynthesis reduces the 3-keto group of acetoacetyl-ACP to generate (*R*)-3-HB-ACP [31]. FabG is one of the components for type II fatty acid synthesis in most bacteria and all plants [45]. The feature of the type II system is the presence of ACP, which shuttles the acyl side chain intermediates as a thioester attached to the terminal sulfhydryl group of a 4′-phosphopantetheinate arm. FabG performs the reduction, leading to an “*R*”-configuration of the acyl side chain of ACP. Biochemical analysis of CQS biosynthesis revealed that 3-HB-CoA is also available as an alternative substrate of 3-HB-ACP [32]. As 3-HB-CoA is synthesized as an “*S*”-configuration by enoyl-CoA hydratase for β-oxidation [46], 3-HB-ACP would be a true precursor for carbazole biosynthesis. In addition to FabG, malonyl-CoA:ACP transferase (FabD) and 4′-phosphopantetheinyl transferase (PPTase), which are necessary for malonyl-ACP supply, are absent in the CQS biosynthetic gene cluster. FabD is absent in most type II PKS gene clusters as well [38]. Therefore, endogenous FabD and PPTase may be recruited from fatty acid biosynthesis in primary metabolism. As mentioned above, considering that CqsB2 is structurally similar to the type II polyketide ARO/CYC, the carbazole-synthesizing machinery and type II polyketide biosynthetic machinery may have an evolutionary relationship.

## 3. Enzymatic Modification of the Carbazole Skeleton

### 3.1. New-Type Carbazole Prenyltransferase

**Figure 6:**
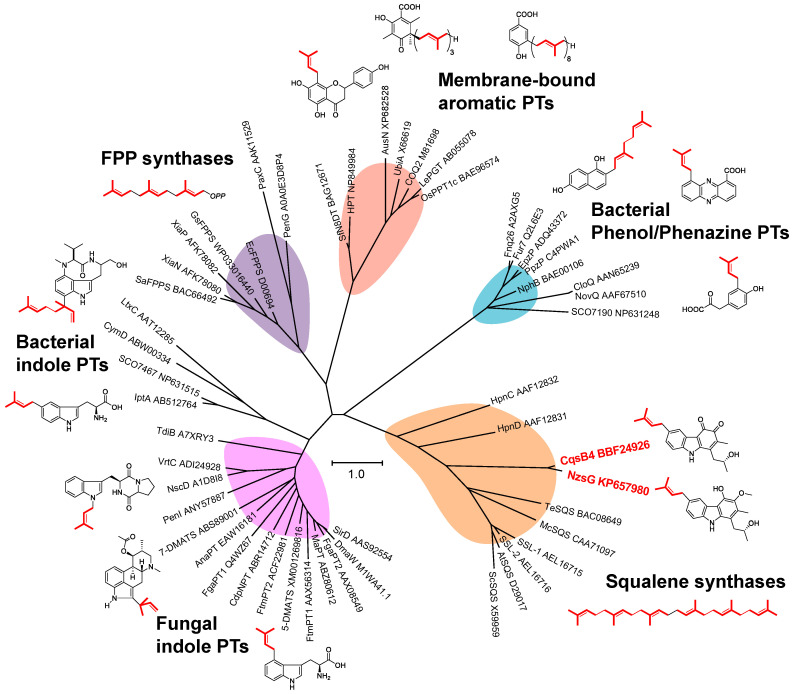
Phylogenetic relationship of prenyltransferases. The accession numbers of each prenyltransferase are included in the tree. The tree was calculated using the maximum likelihood method by MEGA7 [47].

Aromatic substrate prenyltransferases (PTs) catalyze the condensation reaction between the prenyl side chain and an electron-rich aromatic ring. Prenyl side chains are appended in a wide variety of bioactive natural products, including amino acids, alkaloids, polyketides and flavonoids, creating natural product hybrids with altered or enhanced bioactivities [48]. There are several types of aromatic prenylation reactions that generate a high diversity of secondary metabolites in bacteria, fungi, and plants. PTs can be categorized depending on their primary sequence similarity and on their substrates as aromatic acceptors for prenylation (i.e., bacterial phenol/phenazine PTs, fungal or bacterial indole PTs, and membrane-bound aromatic PTs) (Figure 6). The subgroup of soluble aromatic or indole PTs contains an (*ααββ*)_4_-(*αββα*) structural motif (named PT-barrel fold); for example, NphB [49], FgaPT2 [50], and CymD [51], designated as ABBA-type PTs. The PT-barrel fold was discovered during structural elucidation of NphB, which is a key enzyme for naphtherpin biosynthesis [49]. Membrane-bound aromatic PTs have been found in plant secondary metabolism (e.g., LePGT [52]) and primary metabolism (e.g., UbiA in ubiquinone biosynthesis [53]). This subfamily contains two highly conserved aspartate-rich motifs, which are important for binding prenyl diphosphates via divalent metals in the isoprenoid synthase family, including farnesyl diphosphate (FPP) synthases and squalene/phytoene synthases. A phytoene-synthase-like carbazole PT, NzsG, was characterized in NZS biosynthetic studies [29]. NzsG uses dimethylallyl diphosphate (DMAPP) as a prenyl donor substrate and catalyzes a transfer reaction of the prenyl moiety of DMAPP to the carbazole nucleus. In CQS biosynthesis, CqsB4 (NzsG homolog) is essential for prenylation in the last reaction of CQS biosynthesis [32]. Both NzsG and CqsB4 contain two aspartate-rich motifs, which are found in squalene/phytoene synthases and are important for binding the prenyl diphosphate substrates. Intriguingly, these carbazole PT enzymes show no sequence similarity to any of the bacterial or fungal aromatic or indole PTs identified to date. The prenylation mechanism catalyzed by the carbazole PTs stands in total contrast to the feature of the ABBA-type PTs, which have no aspartate rich motifs [54]. Phylogenetic analysis indicated that NzsG and CqsB4 form the same branch with squalene synthases, instead of all the known aromatic or indole PTs, implying that carbazole PTs are a new type of emerging PT family (Figure 6). A biochemical assay of NzsG revealed that there was no turnover when NzsG was incubated with C_5_ DMAPP or C_15_ farnesyl diphosphate and some indole derivatives or other tricyclic molecules, suggesting that the substrate specificity of carbazole PTs is narrow. According to some reports, the prenyl moieties in prenylated aromatic compounds are often important for biological properties [55]. Therefore, further investigation on the prenylation mechanism catalyzed by carbazole PTs would provide crucial information to utilize carbazole PTs to produce valuable prenylated molecules. Crystal structures of phytoene synthase-like carbazole PTs coupled with in vitro assays would provide a basis for understanding and potentially manipulating the regiospecific prenylation of carbazole skeletons using the carbazole PTs.

### 3.2. Isopentenyl Diphosphate Isomerase

In silico analysis revealed that there is an isopentenyl diphosphate (IPP) isomerase in the biosynthetic gene clusters of both CQS and NZS. CqsB8 and NzsC show high homology to type 1 IPP isomerase, which catalyzes the mutual conversions between the relatively unreactive IPP and the more reactive electrophile DMAPP. CqsB8 and NzsC could function as DMAPP suppliers for the prenylation of the carbazole nucleus in the biosynthesis of CQS and NZC, respectively. There are two types of IPP isomerases: type 1 for divalent metal-dependent enzymes and type 2 for Mg^2+^- and FMN-dependent enzymes. Type 1 IPP isomerase is essential for the biosynthesis of the isoprenoid units, IPP and DMAPP, in the mevalonate (MVA) pathway [56]. Type 2 IPP isomerase was identified in pathogenic Gram-positive bacteria and archaea possessing the MVA pathway [57]. Although most bacteria utilize the methyl erythritol phosphate (MEP) pathway, which can simultaneously synthesize both IPP and DAMPP, unlike the MVA pathway, some of these organisms also have type 1 IPP isomerase or type 2 IPP isomerase or both of them. For instance, *Streptomyces* sp. strain CL190, which uses the MEP pathway and the MVA pathway, possesses both type 1 and type 2 IPP isomerases [58]. As the prenyl moiety of CQS is derived from the MEP pathway, the CQS-producing strain *S. exfoliatus* 2419-SVT2 presumably uses only the MEP pathway for IPP and DMAPP biosynthesis [27]. An additional type 1 IPP isomerase, designated Cs-5479, was found in the CQS-producing strain [59]. Cs-5479 could be an enzyme for primary metabolism, while CqsB8 may be a CQS biosynthesis-specific enzyme in *S. exfoliatus* 2419-SVT2. In fact, disruption of the *cqsB8* gene resulted in a significant decrease in CQS production [59]. Thus, the control of DMAPP supply by multiple copies of IPP isomerase may contribute to the efficient production of prenylated secondary metabolites. Differences in biochemical properties between CqsB8 and Cs-5479 would be an interesting subject of study.

### 3.3. Biotransformation of Carbazole Derivatives

**Figure 7:**
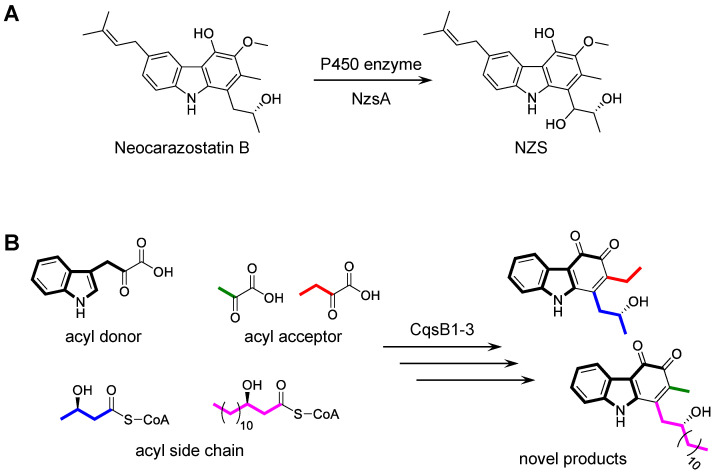
(**A**) Hydroxylation of neocarazostatin B. (**B**) Production of novel carbazole analogs.

Three NZS biosynthetic enzymes, the P450 hydroxylase NzsA, the ThDP-dependent enzyme NzsH, and the KASIII enzyme NzsF, were investigated regarding their substrate specificities. NzsA is responsible for the installation of the hydroxyl group at C-11 of neocarazostatin B to generate NZS (Figure 7A) [29]. When NzsA was incubated with (*R*)-streptoverticillin and precarazostatin, no new products were formed. In studies on the total synthesis of carquinostatin A and lavanduquinocin, an asymmetric synthesis of the core carbazole structures, 6-desprenyl-carquinostatin and 6-descycloavandulyl-lavanduquinocin, was established using lipase QLM (Meito Sangyo Co., Ltd.) and lipase PS (Amano Enzyme Inc.), which catalyze enantioselective acetylation of the hydroxy group on the *para*-alkyl side chain [60]. Of the lipases, lipase QLM showed high enantioselectivities to the racemic alcohol in a key carbazole intermediate during the total synthesis. NzsH mediates the biotransformation of IPA and 2-oxobutyrate only, although other biochemically characterized ThDP-dependent enzymes have broad substrate specificity [30]. Investigations of the substrate tolerance of NzsF showed that the KASIII enzyme accepts propionyl-CoA as the acyl side chain primer [31]. Chemoenzymatic synthesis of carbazole derivatives was performed by CqsB1, 2, and 3 (NzsJ, NzsI, and NzsH homologs) and led to the production of novel carbazoles (Figure 7B) [32]. The analysis of the substrate specificity suggested that CqsB1 and CqsB2 accept only the acyl-CoA or acyl-ACP substrates with a β-hydroxy group, including 3-HB-CoA and 3-hydroxymyristyl-CoA, suggesting that the carbazole biosynthetic enzymes strictly recognize the β-hydroxy group on the acyl substrate. In contrast, in the biosynthesis of carbazoquinocins and carbazomycins, CqsB1-like enzymes likely accept only non-β-hydroxyl acyl substrates such as acyl-ACP and acetyl-ACP, respectively.

## 4. Conserved Gene Clusters Distributed in Bacteria

**Figure 8:**
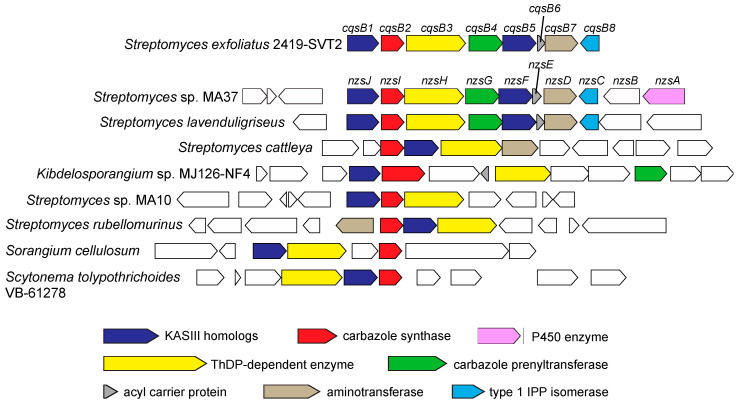
Distribution of homologs of biosynthetic genes for the carbazole skeleton among bacteria.

Bioinformatics analysis showed that the putative biosynthetic gene clusters consisting of the NzsJ, NzsI, and NzsH (CqsB1, CqsB2, and CqsB3 for CQS biosynthesis) homologues are conserved in the genomes of some bacteria, including the Gram-positive actinomycetes, *Streptomyces cattleya*, the soil-dwelling Gram-negative myxobacterium *Sorangium cellulosum,* and the blue green algae *Scytonema tolypothrichoides* (Figure 8) [36]. Multiple Expectation Maximization for Motif Elicitation (MEME) prediction [61] revealed that the NzsJ homologues contain the catalytic residues and the NzsI homologues share highly conserved motifs, indicating that these homologues catalyze the same chemical reactions as NzsJ and NzsI; sequential alkylation and cyclization are catalyzed by NzsJ and NzsI for the formation of carbazole ring, respectively. Furthermore, the conserved gene clusters containing these three homologues are composed of additional putative tailoring enzymes, which contribute to the modification of the carbazole skeleton and/or the acyl side chain. These genomic analyses uncover the wide distribution of the carbazole-synthesizing machinery in nature, facilitating the discovery of uncharacterized carbazole-type secondary metabolites.

## 5. Conclusions

Herein, this review mainly describes the total biosynthetic mechanism for a carbazole backbone catalyzed by several enzymes, especially carbazole synthase, which is a key step in the formation of a carbazole nucleus. The tailoring steps and chemoenzymatic studies of the carbazole skeleton pave the way for engineering carbazole-synthesizing enzymes to establish a method to design a novel carbazole skeleton. Due to the lack of experimental data with regard to other carbazole metabolites derived from higher plants and other microorganisms, biosynthetic studies of these compounds are still limited. Further investigation of other carbazole biosynthesis pathways would provide crucial information about the biogenetic features of these compounds and the unique reactions catalyzed by KASIII-like enzymes and carbazole synthases. Such investigation would facilitate the discovery of new carbazole metabolites.
